# Supplementary figures and images for: Ultraviolet disinfection impacts the microbial community composition and function of treated wastewater effluent and the receiving urban river
Source: PeerJ. 2019 Aug 6;7:e7455. doi: 10.7717/peerj.7455 (PMC6688595; doi:10.7717/peerj.7455)

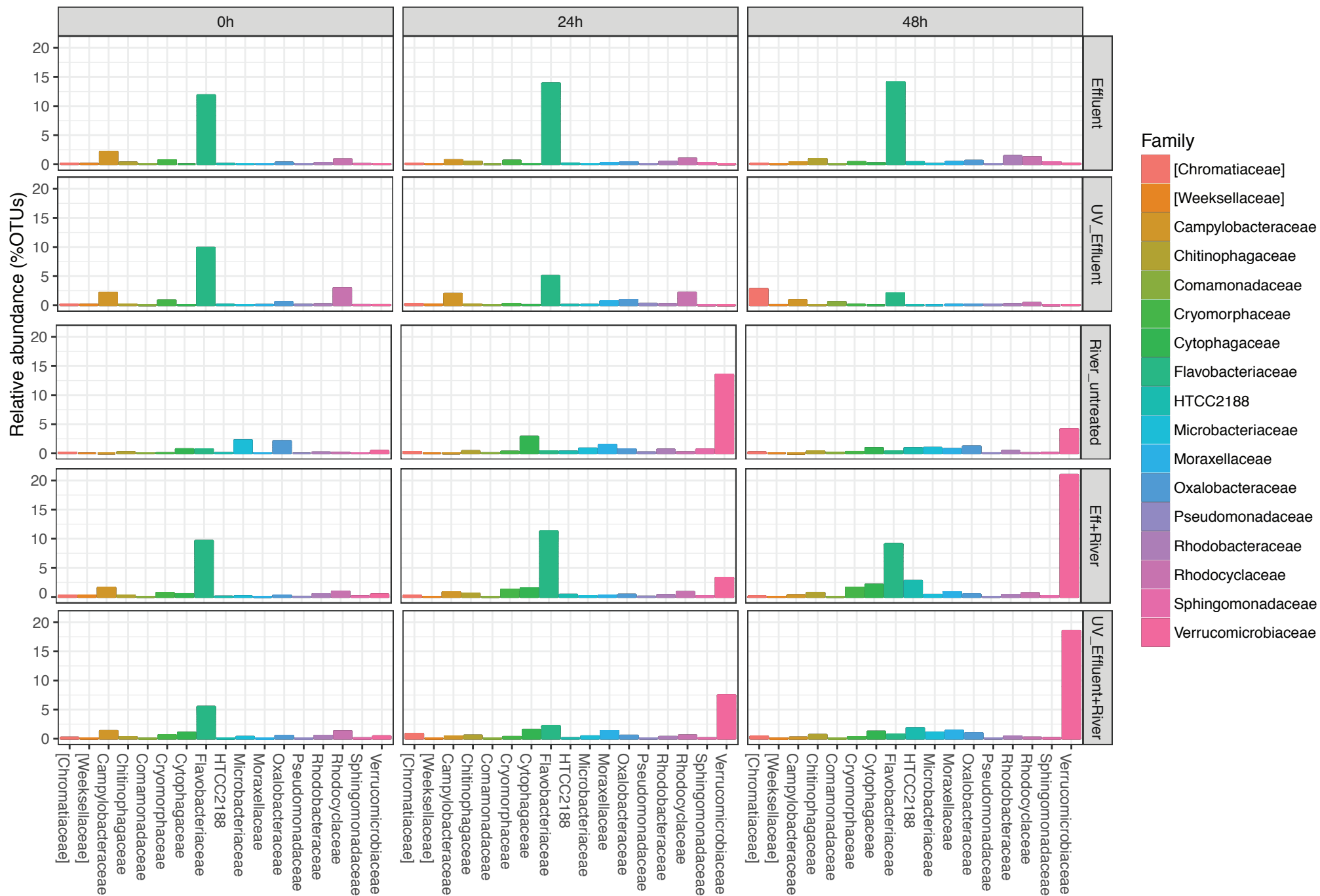

Supplement: Figure S1 — Relative abundance refers to percentage of the OTUs attributed to each family with respect to all OTUs from each sample, including those that were unclassified. Only families represented by a total of >0.1% of OTUs across all samples are shown. The five sample types are separated vertically by treatment (top two are effluent only and bottom three are river water or river with added effluent) and horizontally by time point. [file peerj-07-7455-s001.pdf]

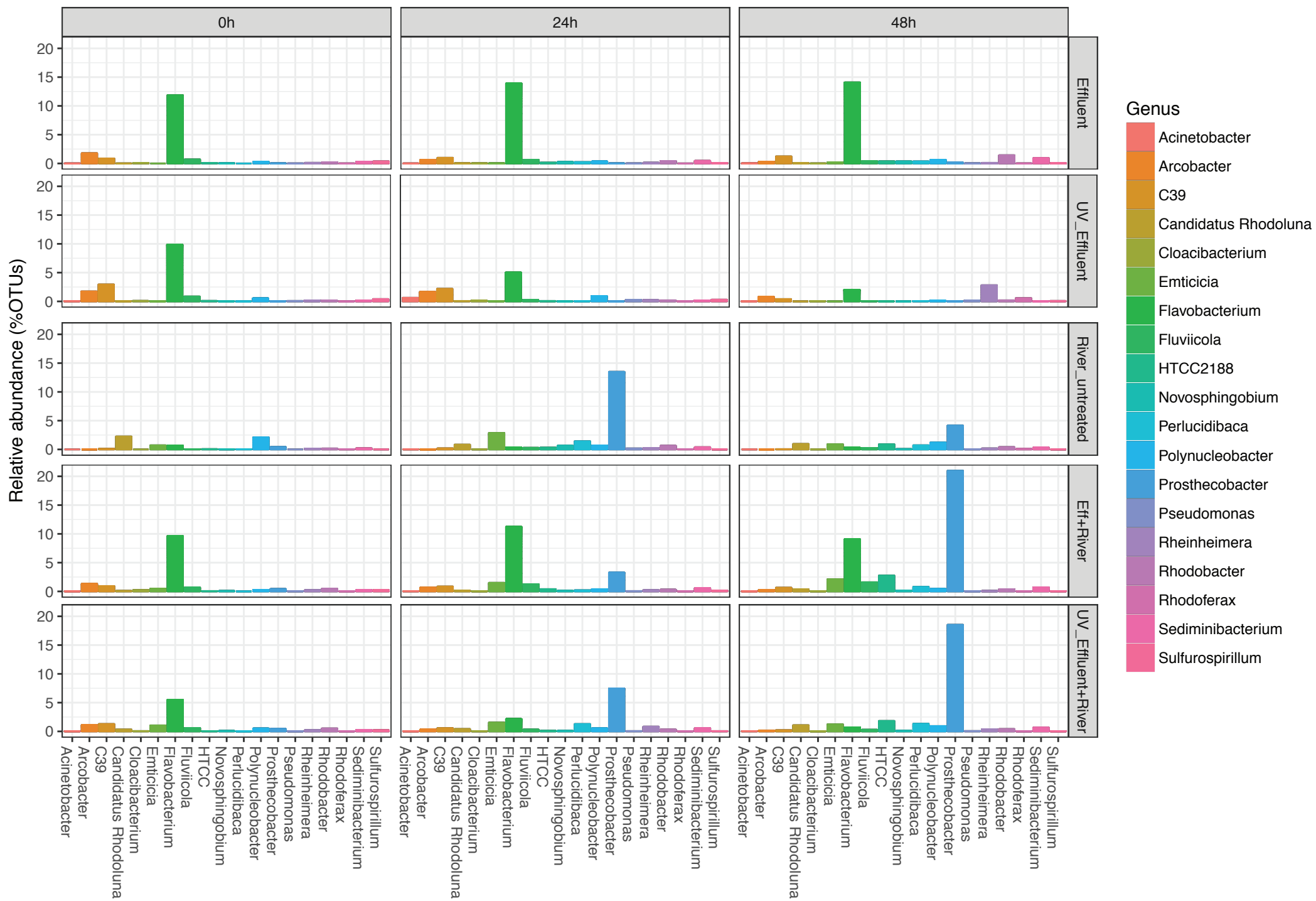

Supplement: Figure S2 — Relative abundance refers to percentage of the OTUs attributed to each genus with respect to all OTUs from each sample, including those that were unclassified. Only genera represented by a total of >0.1% of OTUs across all samples are shown. The five sample types are separated vertically by treatment (top two are effluent only and bottom three are river water or river with added effluent) and horizontally by time point. [file peerj-07-7455-s002.pdf]

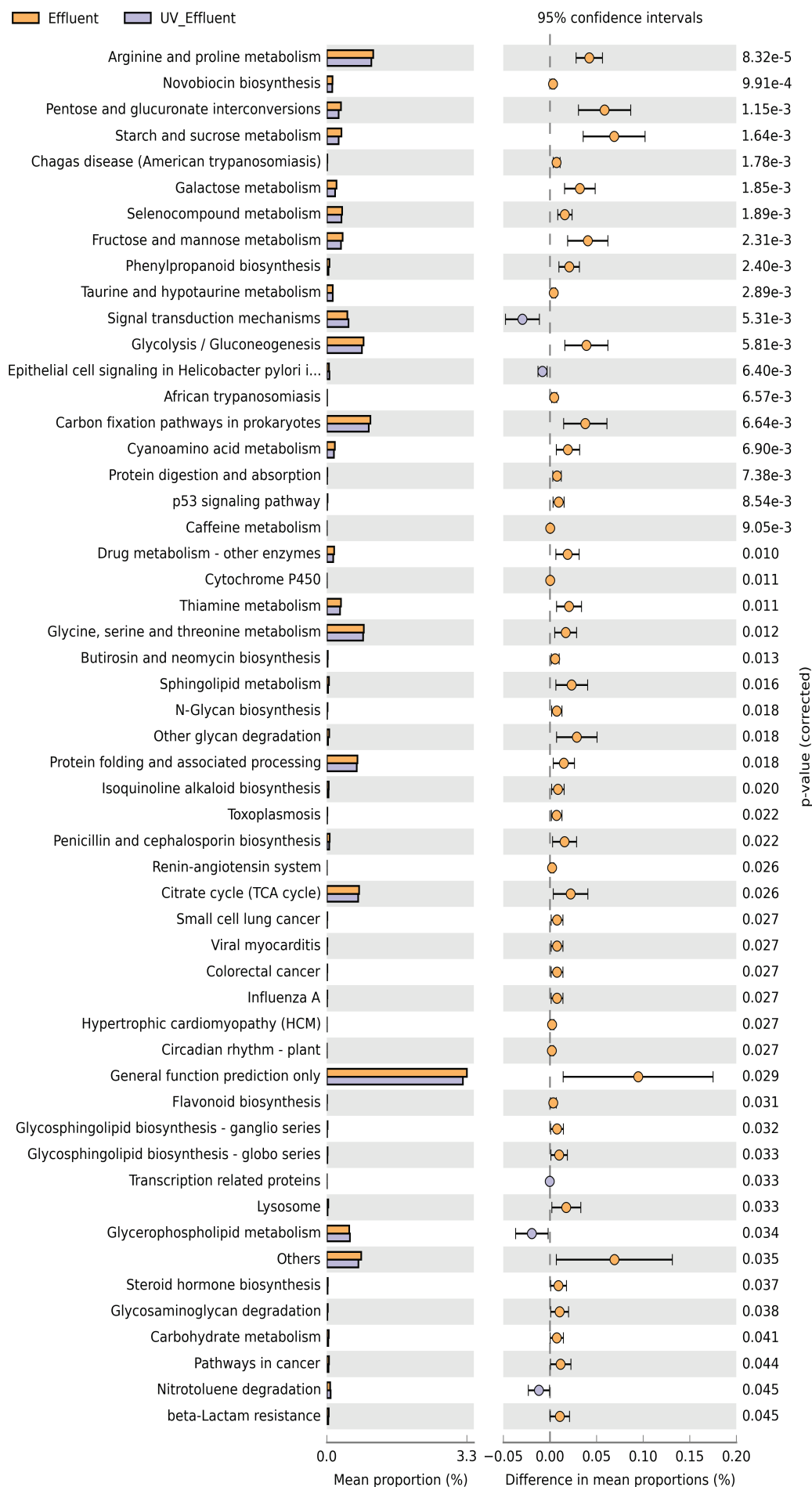

Supplement: Figure S3 — Functions are shown at hierarchical level 3 based on the Kyoto Encyclopedia of Genes and Genomes (KEGG) Orthology (KO) database. Welch’s t-test was used to calculate variance between untreated vs. UV-treated effluent samples, all time points combined. The P-values and 95% confidence intervals for the total variance of the two groups areindicated for each function. [file peerj-07-7455-s003.pdf]
